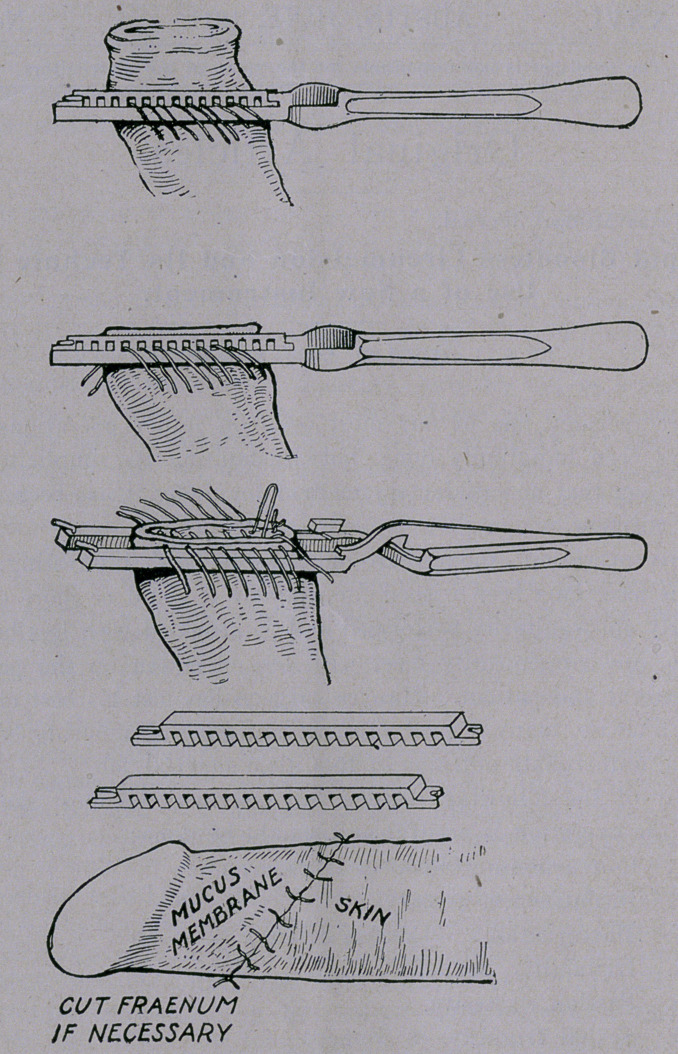# Rapid Bloodless Circumcision and Its Technic by Use of a New Instrument

**Published:** 1911-06

**Authors:** S. L. Kistler

**Affiliations:** Los Angeles, Cal.


### TEXAS MEDICAL JOURNAL.

Established July, 1885

F. E. DANIEL, M. D., - - - - - - Editor, Publisher and Proprietor

Published Monthly.—Subscription $1.00 a Year.

VOL. XXVI AUSTIN, JUNE, 1911. No. 12

The publisher is not responsible for the views of the contributors.

### Original Articles.

For Texas Medical Journal.

### Rapid Bloodless Circumcision and Its Technic by Use of a New Instrument.

BY S. L. KISTLER, LOS ANGELES, CAL.

Circumcision, one of our most common minor operations, has always been bunglingly done, notwithstanding its simplicity, for the reason that no satisfactory method for doing it has been available, till now.

This operation, though an old one, is still interesting, and means have long been wished for whereby it could be more easily, quickly and sanitarily performed and be attended with less hemorrhage, and consequently meet with less objection on the part of the parent and patient. Many a surgeon has lost his best clients, and, likewise, many a good prospect has gone glimmering because of the unfortunate outcome of this little surgical job.

Any of the following reasons will convince the most skeptical that the operation is needful and should be done:

1. Convulsions. (Reduces tendency.)
2. Incontinence, urinary.
3. Masturbation. (Lessens desire.)
4. Irritability, sexual.
5. Passion, excessive.
6. Sexual frigidity, in women.
7. Premature ejaculation. (In male.)
8. Impotency, in men of all ages, especially old men.
9. Spermatorrhea.
10. Venereal disease—not so easily contracted by men—thus fewer cases of pelvic disease in women and consequently fewer belly cases to operate on.

11. Mucous membrane conserved, consequently nerve stimulation better than from ordinary operation.

12. Hygienic.

A casual examination of the cuts which follow will make apparent to the physician the ease with which this operation can be done by use of this instrument. It requires only one-quarter of time that other methods need, is practically bloodless, as all ligatures are uniformly placed and tied before the clamp is removed.

(These cuts are explanatory and show the steps to be followed to completion of work ready for dressing. Cuts about one-half size. Last cut shows operation completed and ready for dressing.)

DIRECTIONS.

1. Use local or general anesthetic.

2. Pull foreskin forward moderately, using spear to extend and hold mucous membrane, if desired.

3. Place "clamp" (meanwhile holding mucous membrane as noted).

4. Insert ligatures, through parts (allowing them to protrude about four inches on each side).

5. Excise foreskin and mucous membrane with scissors.

6. Remove parts (leaving "clamp" blades in position; ligatures being in place.) Now, pull up center of end ligature (by taking hold of it intermediate the walls of mucous membrane) to a height of, say, two inches; cut loop in center and tie on respective sides. So continue until all ligatures are tied. Then place a ligature anteriorly, also one posteriorly, if desired.

7. Three-day, catgut sutures are suggested.

8. Remove "clamp," push tissue over corona, apply dressing and treat as usual.

9. An antiseptic oil dressing is recommended.

10. Adhered cases require splitting on dorsal aspect in order to break down adhesions. Then apply "clamp" and operate as above directed (or split foreskin on both dorsal and inferior aspect and operate one side at a time; no ligatures to pull up; only skin and mucosa to unite).

10. As is evident, a longer portion of the mucous membrane and a comparative shorter portion of skin is obtained by this operation than by the one generally performed.

12. Females are operated on one side at a time. If parts are too long for removal by a single application of the "clamp," you will apply it twice in succession, as is obvious.

A better result is obtained by this operation than by the one usually done, as too much mucous membrane is usually removed and too little skin, whereas the use of this instrument requires that more mucous membrane be left. There is practically no blood, no anxiety, no misgivings as to results, and the work is done quickly and easily.

Many females need circumcision, and the operation is more easily performed than in the male. The clamp is applied singly to such elongated or hypertrophied parts as it is desired to remove, ligatures inserted, part excised, upper part of blades removed, ligatures tied without necessity of pulling up loops, etc., clamp removed and dressing applied.

I have never tried this instrument for protruding pile tumors (of considerable length), but it occurs to me that a beautiful operation could be performed by its use, for the reason that double ligatures could be inserted through the vein posterior to the sacculated portion, and then the set of ligatures used for the vein could be withdrawn from the skin; thus the vein could be tied separately and the skin then closed with the other set of ligatures, loosely tied, which would incidentally hold the vein in close contact with the skin.

The features of my clamp are the notched, removable portion of the blades and the smooth surface along which to remove the redundant tissue without danger of cutting any of the ligatures which have been inserted, thus leaving just the margin required to hold the stitches securely, after which the removal of the loose portions of the blades leave the sutures free for rapid tying, while the remainder of the instrument is still in place.

## Figures and Tables

**Figure f1:**